# Spanish electoral archive. SEA database

**DOI:** 10.1038/s41597-021-00975-y

**Published:** 2021-07-28

**Authors:** Virgilio Pérez, Cristina Aybar, Jose M. Pavía

**Affiliations:** 1 GIPEyOP, Área de Métodos Cuantitativos, Departamento de Economía Aplicada, Facultat d’Economía, Universitat de Valencia, Valencia, Spain; 2 GIPEyOP, UMICCS, Área de Métodos Cuantitativos, Departamento de Economía Aplicada, Facultat d’Economía, Universitat de Valencia, Valencia, Spain

**Keywords:** Sociology, Society, Government, History, Politics

## Abstract

This paper introduces the SEA database (acronym for Spanish Electoral Archive). SEA brings together the most complete public repository available to date on Spanish election outcomes. SEA holds all the results recorded from the electoral processes of General (1979–2019), Regional (1989–2021), Local (1979–2019) and European Parliamentary (1987–2019) elections held in Spain since the restoration of democracy in the late 70 s, in addition to other data sets with electoral content. The data are offered for free and is presented in a homogeneous and friendly format. Most of the databases are available for download with data from various electoral levels, including from the ballot box level. This paper details how the information is organized, what the main variables are on offer for each election, which processes were applied to the data for their homogenization, and discusses future areas of work. This data has many applications, for example, as inputs in election prediction models and in ecological inference algorithms, to study determinants of turnout or voting, or for defining marketing strategies.

## Abstract (summary)

**Table:** 

Measurement(s)	votes
Technology Type(s)	Poll • digital curation
Sample Characteristic - Environment	political entity
Sample Characteristic - Location	Kingdom of Spain

Machine-accessible metadata file describing the reported data: 10.6084/m9.figshare.14674065

## Background & Summary

In an open, modern, transparent society, access to detailed and reliable information is essential. Basic data should not only be accessible, but should also be available in user-friendly formats. In democratic systems, electoral results represent the manifestation of the popular will. Publication of these results is essential to give the system the necessary legitimacy. In the case of Spain, despite the so-called Law of Transparency^1^, gaining access to detailed electoral data can sometimes be convoluted and, even once access is gained, the format of the data often needs to be edited and homogenized before being usable.

Election data is a valuable source of information for answering a multitude of research questions, profiling citizens, and implementing business models. Electoral behaviour varies depending on the region, the province, the municipality, the neighbourhood, the district, the census section or the ballot box. Having detailed results for small areas (for example, at the municipal, section or polling station level) is relevant for researchers, analysts and professionals. For example, the information is useful for the definition of electoral marketing strategies^2^, for geographic analysis^3^, to generate estimates with data from pre-electoral surveys using models of small areas^4^, to improve the predictions of exit polls^5–7^ and quick counts^8^, and for use in spatial or spatio-temporal electoral models^9^. The previous examples are not exhaustive; the data can also be used, among other things, as inputs in ecological inference algorithms^10–12^, in electoral night prediction models^13,14^, to study determinants of participation or voting^15,16^, or for redrawing of the electoral spaces^17,18^.

In the case of electoral processes organized by, and the responsibility of, the government of Spain (elections to Congress and the Senate, municipal elections, European Parliamentary elections, elections to councils or referendums), the results are available for direct download in the “*Área de Descargas*” from the website of the Spanish Ministry of the Interior. For most elections, data available on this website can be as detailed as at the ballot box level, the minimum level of aggregation in which votes are counted and published in Spain.

For most citizens and a number of social scientists, however, these files are nothing more than strange hieroglyphics that are difficult to read. In general, these agents do not have the necessary computer skills to transform files, i.e., turn data into valuable information that they can analyse. The files for download are composed of several ASCII files (with fixed positions and long-table structures) filled with numerical codes that label the information. Metadata documents are necessary in order to interpret and transform the content of ASCII files into more intuitive formats.

In the case of regional elections, the access to the data is even more problematic. There are regions (autonomous communities) that have active web pages in which they offer files of the results (each file with its own format and characteristics). Others make the data available briefly to the public, then make it disappear without prior notice, or go one step further by making the page that hosted it or the agency in charge disappear (see examples in Online-only Table 1). There are autonomous communities that never even make their detailed electoral results public, and getting access to this information is, at times, far from easy. It becomes even harder the further removed the date is from the election date, or when there has been a change of government or as a consequence of a misunderstood sense of ownership of data. Finding out who to contact to make the request, be it person/service/body, is difficult but, once done, actually getting the data can take months, even years.

Undoubtedly, many agents would find it useful to have a centralized location where they can access, in a homogeneous and friendly format, all the electoral data available for Spain. The research group on Electoral Processes and Public Opinion of the University of Valencia (GIPEyOP) has been undertaking this task for a number of years (sometimes driven by our own needs), collecting and homogenizing the information associated with the main electoral processes held in Spain. All the data collected by GIPEyOP http://gipeyop.uv.es/ are available in an open, free and homogeneous format in the SEA database (acronym for: Spanish Electoral Archive) and are accessible through the web pages http://sea.uv.es/gipeyop/sea.html and https://dataverse.harvard.edu/dataverse/SEA. This article describes the structure and variables that can be found in the different files that make up SEA, and reports on the enrichment and improvement processes that GIPEyOP is undertaking to expand the quality and scope of the data.

## Methods

The SEA Database has been developed from processing and standardizing official (provisional and proclaimed) outcomes available from different sources and in different formats. The primary data sources and the processing performed are detailed below, grouped by type of election.

### General elections

In the case of the General Elections, SEA offers for download all the results corresponding to the elections to the Congress of Deputies held in Spain since the restoration of democracy. This represents a total of 15 elections at the time of writing this article: from the elections held in June 1977 to the last of November 2019. The primary source of information for the data of these elections is the Ministry of the Interior of Spain in the block “*Extracción de datos*” from the “*Área de Descargas*” section: http://www.infoelectoral.mir.es/infoelectoral/min/areaDescarga.html.

In each electoral process, the Spanish Ministry of the Interior offers, for download, data at the maximum level of detail available (usually at the ballot box level). The download is offered in the form of a compressed file (zip) containing 12 files: 10 ASCII files, with a DAT extension, 1 Microsoft Word file, with a doc extension, and a final rich text file, with an rft extension. The 10 DAT files have a fixed length, text format and a constant structure, which facilitates the programming load for their reading and subsequent compilation. The doc and rft files are metadata files that help with understanding the content and structure of the information in DAT files.

Specifically, there are four DAT files that must be processed to get some of the variables of interest listed in the next section: the 03xxyymm.DAT file, from which information on the candidatures (codes, acronyms and party names) is obtained; the 05xxyymm.DAT file, which offers us information on the spatial identifiers of the voting units (codes and names); the 09xxyymm.DAT file, which contains, among other variables, census information and information on blank and null votes; and the file 10xxyymm.DAT that collects the votes per candidacy. In these file names ‘xx’ tells us about the type of electoral process (for example, when xx = 02, this signifies elections to the Congress of Deputies, xx = 04 Municipal elections and xx = 05 elections to the European Parliament), and ‘yymm’ the year and the month when the election was held.

All the data processing to transform the unfriendly data formats and metadata of the Ministry of the Interior into structured data workbooks has been done using ad-hoc scripts in R^19^, executed through RStudio^20^. Once all the information contained in files 03, 05, 09 and 10 has been extracted, the information for the higher aggregation scopes is generated (again with ad-hoc R scripts).

### Regional elections

The data processing associated with the Regional (Autonomous) Elections presents a further complexity to that of other elections, a complexity that resides in its multiple sources of information. Management of the electoral processes in each autonomous community is self-governing and does not depend on the Ministry of the Interior. The difficulty is that not all autonomous governments offer the results on public platforms. In some cases, it is the Statistical Institutes of the corresponding autonomous community which offer the information, in other cases this information is not available. The procedure to follow when the information is not public, or is not easily located, is to contact the person responsible for the electoral processes of the corresponding administration. Finding the right contact, however, can be a lengthy, and not always successful, process.

Sometimes the results are obtained after being referred to the company that was hired to manage the entire counting process. Being dependent on the company contracted by each autonomous government can be problematic because the company chosen may change following an election win or after changes of government. This means there is a possibility that the links that we offer in our processed files as the primary source of data may become obsolete. Likewise, there have been autonomous governments that have provided us with information directly from a representative of their administration, so in these cases we cannot offer the link to the original raw data. Online-only Table 1 offers information on the links of the autonomous communities and their current status.

The content of Online-only Table 1 is a clear reflection of the difficulties involved in merely obtaining electoral results at the autonomous community level and its heterogeneous level of transparency in relation to electoral data. The information presented in Online-only Table 1 gives an idea of the haphazard approach to offering access to data and the problems that have arisen in the process of collecting electoral information through regional practices, difficulties which have made the process of creating SEA all the more challenging, but necessary.

### Local elections

The primary source of information of the results of municipal elections is the same as that of the European and General elections: the Ministry of the Interior. Municipal elections are held every 4 years. To date, these elections have been held in the following years: 1979, 1983, 1987, 1991, 1995, 1999, 2003, 2007, 2011, 2015 and 2019. SEA (the Ministry of the Interior) has completed information (that is, from ballot box level) since the 1987 elections. For the first two elections, 1979 and 1983, the results are only available since municipal (local) level.

The advantage of having the Ministry of the Interior as a source of information is that it always presents the same structure, the same text files, and this facilitates data processing, which is why similar procedures to those described above were used. The difficulty, in this case, lies in the fact that in each municipality different candidacies are presented, and extra programming efforts are required to resolve this issue.

### European Parliamentary elections

The electoral years corresponding to European Parliamentary elections are: 1987, 1989, 1994, 1999, 2004, 2009, 2014 and 2019. For all of these there is raw data per ballot box and the source of information is, as in the other national electoral processes, the Ministry of the Interior. The data processing is similar to those described for the general elections, although with some particular features.

In these elections, despite the fact that Spain represents a single constituency, a large number of different candidacies are usually presented, which makes it difficult to achieve representation. The electoral law^21^, however, allows different candidates to form coalitions (groupings) that must be considered together when distributing MEPs. In this way, the votes received by candidacies that use different acronyms and party names in different territories are added together before applying the electoral rules that transform the votes into representatives^22^. This idiosyncrasy of these elections has made it expedient to build two files for each election: a file with disaggregated voting data for each of the candidacies presented, and another, more reduced and manageable, which facilitates the processing and interpretation of the data, obtained by merging the parties that are part of the same coalition when it comes to distributing MEPs, although they are presented under different names in different territories.

### Other sources of data

In addition to purely electoral results, SEA also collates more heterogeneous information in a dataset called “Spanish Elections-Others” (hereinafter “Others”), which, as it grows in the coming years, may lead to new data sets that will be need to be properly documented and communicated. “Others” includes examples of tailor-made compilations, the result of linking various electoral files or linking an electoral file with other sources of information, such as: cartographic files (shape), which contain the geographical limits of the spatial divisions, or population register data files, both available from the scope of the census sections.

It should be noted that correspondence between census sections of the cartographic, electoral and register files, of the same year, do not always coincide^23,24^ and, moreover, they vary over time^25^, so any combination of these sources requires previous processing. Details of the processing should be offered as additional information so that anyone using such files is aware of the limitations, and the hypotheses, of the information contained in them. At the time of writing, “Others” only offers a few examples of these uses. These are examples that are characterized by presenting a direct correspondence between sections, for which the simplest version of the proposals made by Pavía-Miralles^14^ has been used to establish correspondences (matching sections). The files available in this section will gradually increase as we automate the processes of matching and, above all, documentation.

The advantage of combining information sources is that it offers more analysis possibilities. For example, based on the combination offered in SEA that includes the results of general elections and the population register of 1 January, 2019, an analyst could apply ecological inference techniques^26^ using covariates, better define the sampling frames in surveys elections^27^, or study the contextual effects that the number and composition of immigration in each census section have on voting^28^.

## Data Records

The SEA Database is available through the Harvard dataverse repository https://dataverse.harvard.edu/dataverse/SEA. It contains 974 files in Microsoft Excel format, as well as some compressed (rar) files. Currently, SEA is structured into 4 thematic datasets, plus a fifth additional data set. The names of the first four data sets identify the typology of elections reflected in the data: “Spanish General Elections”^29^, “Spanish Regional Elections”^30^, “Spanish Local Elections”^31^ and “Spanish European Elections”^32^. The fifth dataset, “Spanish Elections - Others”^33^, is more heterogeneous. In the data sets of regional elections and municipal elections, a folder structure has also been used to facilitate access to the files. The autonomous community (regional) elections data set is made up of nineteen folders, one for each of the seventeen autonomous communities (CCAA) plus two additional ones for the autonomous cities of Ceuta and Melilla. Each of the folders is identified with the name of the corresponding region (city). Similarly, the municipal elections data set is (currently) composed of eleven folders, one for each of the years in which elections of this type have been held (every four years, from 1979 to 2019). The use of *Tree View* is recommended to navigate these data sets, as each folder contains dozens of files.

The files for download are generally Microsoft Excel workbooks, with a couple of exceptions: the compressed files available in the municipal elections data set, which contain the Excel workbooks of the voting results corresponding to local elections in different areas (more details later), and some of the files for download to the “Others” data set. In this last data set, all the files corresponding to a specific electoral cartography (including the shape files that define the polygonal lines associated with the census sections and the associated dbf database of electoral outcomes) are offered compressed into a single downloadable file. The description that follows mainly focuses on the content that can be found in the Excel workbooks available in the four main data sets. These workbooks contain the official and provisional election results (recorded during the count), and constitute the main content of SEA.

### Overview of the data files

Each Microsoft Excel workbook contains detailed results of a single election and can contain up to ten spreadsheets, with titles that indicate their contents. An initial spreadsheet (PREVIEW) has been included in each workbook to inform users that the data cannot be previewed in dataverse, indicating the need to download the files beforehand. The spreadsheets that can be found in each workbook, and an explanation of their content, are detailed below.

**Fig. 1:**
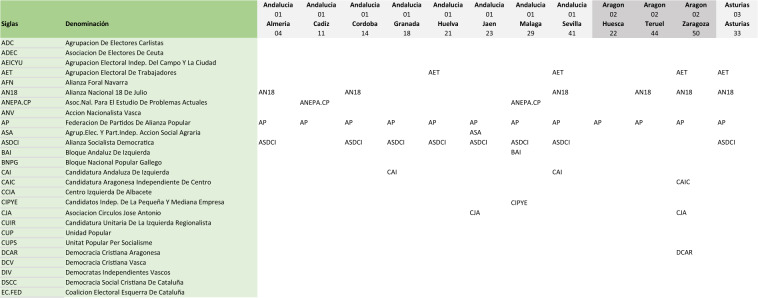
Example of excerpt of metadata contained in a CANDIDADURAS spreadsheet. The first column shows the acronym of the candidacy. The second column offers the full name of the candidacy. The following columns give the acronyms used by each candidacy in each territory. In the case that a candidacy does not compete in a territory the corresponding cell is left blank.

- LEEME (Readme, in Spanish): This spreadsheet indicates the primary source of the raw, provisional and official data, as well as the formula proposed to cite the data contained in the file. Given that the effort to homogenize, maintain and feed this electoral repository is onerous, in exchange for offering the data openly and free of charge, we at GIPEyOP ask all those analysts and researchers who make use of any of the databases to cite us in their publications.
- LICENCIA (Licence, in Spanish): This spreadsheet collects information on the license of use of the data. License CC-BY 4.0.
- CANDIDATURAS (candidacies, in Spanish): This spreadsheet provides details of all the candidacies that participated in the election referred to in the workbook and whose voting figures can be found in the results spreadsheets. This spreadsheet contains information presented in at least two columns, with the acronym of the candidacies in the first column and the name of the candidacy in the second. If the electoral space is divided into constituencies, the following columns, headed by the constituency identifiers (for example, INE code of province and name of province), contain the information with the acronym with which each candidacy competed in each constituency: see the example in Fig. 1.

- MESAS (Ballot boxes, in Spanish): This spreadsheet offers, among other variables, the results recorded at each ballot box.
- SECCIONES (Census sections, in Spanish): This spreadsheet offers, among other variables, the results recorded in each census section, obtained directly from the source when there is no data from ballot boxes or by aggregating the results by ballot boxes that make up each section.
- DISTRITOS (Local districts, in Spanish): This spreadsheet offers, among other variables, the results recorded in each district, obtained by aggregating the results by section that make up each district.
- MUNICIPIOS (Municipalities, in Spanish): This spreadsheet offers, among other variables, the results recorded in each municipality, obtained directly from the source when more disaggregated data are not available or by aggregating the results by district that make up each municipality. In the case of municipal elections, this tab is divided into MUNICIPIOS (PROVISIONAL) and MUNICIPIOS (OFICIAL), corresponding to the provisional data registered in each municipality, obtained by aggregating the results by districts, and to the official data obtained from the official source.
- PROVINCIAS (PROVISIONALES) (Provinces, in Spanish - provisional results): This spreadsheet—available for the workbooks of the datasets of general, regional and European elections—offers, among other variables, the provisional results registered in each province, obtained by aggregating the results by municipalities that make up each province (constituency or island). In the event that the natural division of the electoral space is by constituency or island, this is the name given to the spreadsheet.
- PROVINCIAS (OFICIALES) (Provinces, in Spanish - official results): This spreadsheet, available for the workbooks of the datasets of general, regional and European elections (in the latter case only for 2019 and 2014), offers, among other variables, the official results recorded in each province. In the event that the natural division of the electoral space is by constituency or island, the information refers to that area and that is the name given to the spreadsheet. The data contained in these spreadsheets has been obtained, in the case of the general and European elections, from the website of the Ministry of the Interior of Spain and, in the case of the regional elections, from the Central Electoral Board.
- CCAA (PROVISIONALES) (Regions, in Spanish - provisional results): This spreadsheet, available for the workbooks of the datasets of general and European elections, offers, among other variables, the provisional raw results recorded in each autonomous community obtained by aggregating the provisional results by provinces (constituencies or islands) that make up each autonomous community.
- CCAA (OFICIALES) (Regions, in Spanish - official results): This spreadsheet, available for the workbooks of the datasets of general and European elections, offers, among other variables, the official results recorded in each autonomous community, obtained by aggregating the official outcomes by provinces (constituencies or islands) that make up each autonomous community.
- INCIDENCIAS (Notes, in Spanish): This spreadsheet collects the incidents found, and the decisions taken to correct the errors found, in the data associated with the corresponding database.

One particular characteristic of the CANDIDADURAS spreadsheet in the general election workbooks is that the party name of the same national candidacy may differ between provinces (see Fig. 1). Elections to the Congress of Deputies can be seen as 52 different elections, although strongly interdependent and correlated^34^, with different candidates in each constituency. For example, the Partido Socialista Obrero Español (PSOE) is called in Catalonia PSC.PSOE (acronym for Partit dels Socialistes de Catalunya - PSOE) while in the Basque Country it is called PSE.PSOE (acronym for Partido Socialista de Euskadi - PSOE) but both would be identified on the results spreadsheets with the generic acronym PSOE.

The internal composition of Excel workbooks described in the previous paragraphs shows the general situation. Later on in the paper, we specify graphically the level of information available for each specific election, since the information has not always been preserved for the lowest levels of aggregation.

### Variables available in the spreadsheets of election outcomes

The number of variables (columns with information) available on each results spreadsheet is very heterogeneous, as it depends on the election, the geographical area to which it refers and, above all, the number of candidacies that competed. For some spreadsheets the number of available variables can exceed one hundred, while for others it may be less than twenty. Each row contains data of a voting unit and each column a variable, being votes to candidacies the more popular variables.

We now focus on briefly describing the variables that are present in almost all spreadsheets that contain poll results. Common variables that can be divided into two subsets: Identifier variables and Result (or numeric) variables.

#### Identifier variables

Each electoral process is held at a moment in time and in a specific place. The identifying variables are variables that collect this information, offering context and enabling the spatial and temporal location of the values associated with other variables. Identifier variables can be divided into temporal variables and spatial variables.

Temporal variables include ANYO, which identifies the year in which the election is held, and MES, which identifies the month of the electoral process. The values of these variables are common in all the rows of the same spreadsheet. The spatial variables are used to identify each polling unit, using the names (code) given to them by the National Institute of Statistics (INE).

The organization of elections is a logistically complex process that requires the intervention of a multitude of agents and must ensure that citizens with the right to vote know where to go to be able to exercise it. The INE is in charge of this task in Spain, informing each citizen with the right to vote of the ballot box (voting table) assigned to him/her and the place (polling station) where this ballot box is located. Each voter is assigned to only one polling station, where he/she can exercise the right to vote on election day.

The voting tables (ballot boxes) are decided as a consequence of dividing the voters registered in the same census section based on the first letter of their first surname. The census sections are created (directly or indirectly by the INE) after dividing (disjointedly) each municipality into small areas which vary in surface area but which contain, as a rule, a maximum of 1500 people with the right to vote in general elections. The census sections of the same municipality can, in turn, be grouped into districts.

The entire Spanish territory is hierarchically divided geographically into autonomous communities (including autonomous cities), provinces, municipalities, districts and census sections. Each of these units is identified with a code and, in the case of the more established administrative units (regions, provinces and municipalities), also with a name. The hierarchically-ordered concatenation of these codes makes it possible to assign a unique code to each ballot box. Each results spreadsheet has a first coding variable (for ballot box, section, district, municipality and provinces spreadsheets, respectively, being denoted cod.mesa, cod.sscc, cod.dist, cod.mun and cod.prov) that uniquely identifies each row of the corresponding spreadsheet. These unique codes are constructed as a concatenation of identifying codes for each of the higher scopes in the hierarchy (see Table 1).

**Table 1:** Identifying variables of voting units.

Variable	Description
COD.CCAA	2-digit code identifying the Autonomous Community. The codes used are those of the Ministry of the Interior; INE codes differ from these for some Autonomous Communities.
CCAA	Denomination of the Autonomous Community
COD.PROV	2-digit INE code identifying the Province.
PROVINCIA	Denomination of the Province
COD.MUN	INE code of 3-digits identifying the Municipality within the province. The code 999 identifies a virtual municipality that includes absent residents with voting rights in the province.
MUNICIPIO	Denomination of the Municipality. Obviously this variable, and the previous one, are not available for spreadsheets that offer results at levels of aggregation higher than municipality level.
DISTRITO	2-digit identifier code of the District within the municipality. Obviously this variable is not available for spreadsheets that offer results at levels of aggregation higher than district level.
SECCIÓN	3-digit identifier code of the Census Section within the district. Obviously, this variable is not available for spreadsheets that offer results at levels of aggregation higher than the census section level.
MESA	Identifying letter of the ballot box within the census section. Obviously this variable is not available for spreadsheets that offer results at aggregation levels higher than the polling station level.

Source: compiled by the authors.

For example, cod.mesa is an alphanumeric code of 13 characters resulting from the concatenation of the code (of the Ministry of the Interior) of the autonomous community (2 digits), and of the INE codes of province (2 digits), municipality (3 digits), district (2 digits), section (3 digits) and ballot box (1 letter). Each ballot box, in each election, therefore has a unique alphanumeric code that identifies it as well as the territorial units *included* in this code: census sections, districts, municipalities, provinces and autonomous communities. It should be noted, however, that in the case of databases corresponding to regional elections, the autonomous community code is omitted.

In addition to the unique identifier code resulting from the concatenation, the rest of the spatial identifier codes and names are also provided to help locate the data in the corresponding row of the data page. We are particularly interested in including the names of the geographical areas. Table 1 presents the description of the spatial variables.

**Fig. 2:**
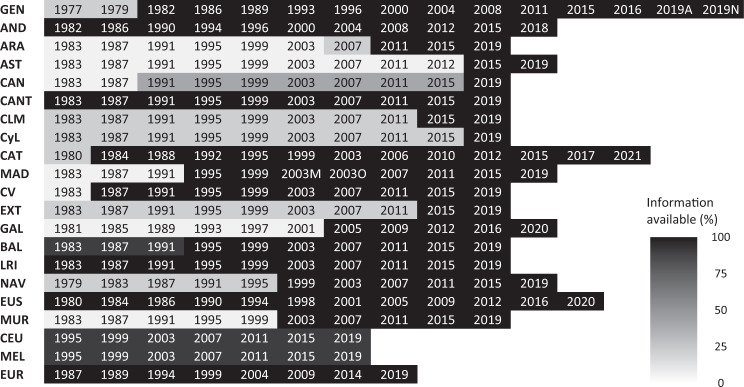
Information available for national (General and European Parliament) and regional elections. The different levels of information available are represented using a descending scale of grey; the lower the tonality, the less information available, with black representing the maximum level of information. The acronyms to identify each of the electoral autonomous communities (cities) are provided in Online-only Table 1. The first row (GEN) refers to general elections, and the last row (EUR) to the European Parliamentary elections. The numbers represent the year that the elections were held.

The level of aggregation available for each of the general elections is detailed in the first row of Fig. 2. Specifically, for all general elections, except those of 1977 and 1979, data are available from ballot box level (the minimum level of disaggregation possible). For 1977 and 1979, data are available from the municipal level. The remaining rows in the figure report on the levels of detail available in each of the regional elections and the last row in the elections to the European Parliament.

#### Results or numerical variables

The temporal and spatial identifying variables tell us the time and the voting unit to which each of the values offered in the Results or Numerical variables corresponds. Table 2 provides a description of the main Results variables.

**Table 2:** Results or Numerical variables.

Variable	Description
CENSO	INE Census. Number of resident electors with the right to vote.
CENSO + CERA	INE Census + CERA census (Absent Residents Electoral Census): Total number of voters with the right to vote.
CENSO.CERE	Census CERE. (Electoral Census of Foreign Residents).
VOT.AVANCE1	First advanced count of voter participation.
VOT.AVANCE2	Second advanced count of voter participation.
BLANCOS	Number of blank votes registered.
NULOS	Number of invalid (null) votes registered.
VOTOS.CANDIDATURAS	Sum of the votes received for the total number of candidacies.
CANDIDATURA_1	Number of votes received by candidacy 1. The corresponding initials appear as the name of the candidacy in each database.
CANDIDATURA_i	Number of votes received by the i-*th* candidacy. The corresponding initials appear in each database as the name of the candidacy.
CANDIDATURA_n	Number of votes received by the n-*th* candidacy. The corresponding initials appear in each database as the name of the candidacy.

Source: compiled by the authors.

For the variables associated with votes for candidacies, the criteria of leaving blank cells has been used in the databases when a certain candidacy did not compete in a certain voting unit and, therefore, could not be voted on. This is different from a zero appearing. If a zero appears in the cell, it means that this candidacy did not obtain any vote in that spatial unit, even though it could have been voted on. The columns of candidacies are arranged in alphabetical order, or from highest to lowest number of votes in the election.

#### Other variables included in the databases

The previously mentioned variables are not the only ones contained in the databases. Depending on the election, in addition to these variables, there are additional variables (both identifying and numerical) that provide complementary and useful information for other analyses. For example, within the identifying variables, we find data spreadsheets that include variables such as CP (the postal code where the voting place, sometimes referred to as polling station, is located), COD.COLE (the code that the INE assigns to the voting place), COLEGIO (the name of the voting place), DIRECCION (the postal address, street and number, of the voting place) or variables such as LETRA_INI and LETRA_FIN, which identify the initial and final letters of the surnames of the people with the right to vote at each table voting, or ISLA or COMARCA, which identify the island or region where the voting unit to which the row refers is located.

Among the additional result variables, we can cite variables such as VOTOS.INTERVENTORES (votes of representatives of political parties not included in the census where they vote, but with the right to vote at the voting table where they are acting as representatives of the candidacies), as well as other variables such as: VOTOS.TOTALES (total of registered votes), VOTOS.ELECTORES (registered votes of voters included in the census lists), ABSTENCIONES (number of registered voters who have not exercised their right to vote) or VALIDOS (valid votes).

Obviously, although the variables in this subsection provide less added value, they may be of interest for some analyses. For example, with the help of the variables COD.COLE and DIRECCION, the percentages of votes registered by polling station could be represented on a map and thus the spatial patterns revealed or geo-statistical techniques^35^ could be applied. Likewise, the variables VOTO.AVANCE1 and VOTO.AVANCE2 could be used to generate predictions of participation at the end of election day, which could be useful in an exit poll^36^.

### More details about the content of the datasets

In the previous sections we have given an overview of the content of the workbooks, mainly focused on the “Spanish General Elections” data set. Next, we describe, in different subsections, differentiating details regarding other data sets.

#### Regional elections

As a rule, the spreadsheets that define the Excel files for each regional election are similar to those for a general election, excluding spreadsheets CCAA (PROVISIONAL) and CCAA (OFFICIAL). There are some exceptions regarding the names of the spreadsheets or their content. Such is the case of Asturias where, instead of provinces, we speak of constituencies (Centre, West and East), and of the Balearic Islands, where the ISLAS spreadsheet refers to Formentera, Ibiza, Mallorca and Menorca, and of the Canary Islands, where the ISLAS spreadsheet contains information on El Hierro, Fuerteventura, Gran Canaria, La Gomera, Lanzarote, La Palma and Tenerife, grouped into two provinces: Las Palmas and Santa Cruz de Tenerife. The Canary databases, therefore, also include the PROVINCES spreadsheets.

**Fig. 3:**
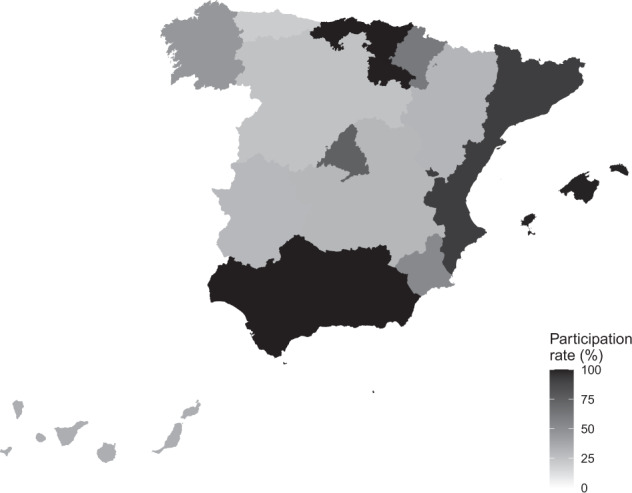
Information available in regional elections. The different levels of information are represented using a descending scale of grey; the lower the tonality, the less information available, with black representing the maximum level of information. The levels of information in each region have been calculated as a percentage of the number of rows available in the data spreadsheets over the total potential of rows that should exist if data relating to all levels of the election were reported. Rounding to one decimal place, the information levels are: Andalucía 99.9%, Islas Baleares 99.2%, Cantabria 99.9%, Ceuta 97.8%, Melilla 97.3%, La Rioja 99.7%, País Vasco 99.9%, Comunitat Valenciana 92.1%, Catalunya 93.6%, Comunidad de Madrid 74.9%, Navarra 61.5%, Murcia 53.2%, Galicia 45.6%, Canarias 34.4%, Aragón 31%, Castilla La Mancha 30.5%, Extremadura 29.6%, Castilla León 25.2% and Asturias 20.8%.

Regarding the depth and level of information that can be found for each of the Autonomous Elections, we also observe a high degree of heterogeneity. As can be seen in Figs. 2 and 3, there are different degrees of information available. The most comprehensive corresponds to a situation where data is available at the ballot box level and, in addition, there is information on CERA voters. In the event that this latter information is available, the fictitious municipality that brings them together has been denoted in the databases by CERAxx, where ‘xx’ represents the code of the corresponding province.

The information available can range from being complete to lacking certain data, such as, data on CERA voters or detailed results by ballot box, census section, municipality, or at worst, by (constituency) province level. The indicators on the levels of information associated with each election, detailed in Figs. 2 and 3, have been calculated as a percentage of the number of rows available in the databases and the potential total of rows. That is, the number of rows that the databases should contain if all ballot boxes reported their votes. Figure 2 provides detailed information for each election. Figure 3 presents, geographically, the global levels of information available from regional elections.

As can be seen, the general tonality of the map in Fig. 3 is not as dark as we would like, since only 7 of the 19 regions (36.8%) have (almost) complete information. The good news is that, at least for the last election, all the autonomous communities have information at ballot box level (see Fig. 2), a positive response that we hope will be maintained in successive elections.

#### Local elections

Spain currently has a total of 8,131 municipalities. In our opinion, attempting to offer detailed, user-friendly information files on each of the elections in each of the municipalities would be impractical. Obviously, this limitation could be remedied by developing a query application that allows the construction, on demand and in real time, from ASCII files of the files requested by an analyst. This application is still being developed, which is part of our future work plan, but in the meantime, in the “Spanish local elections” data set, we have decided to offer already prepared information on the results of local elections for the municipalities that would foreseeably arouse more interest. This includes a total of 52 provincial capitals and 2 CCAA capitals, Mérida and Santiago de Compostela, and 14 of the most populated large non-capital municipalities, excluding those already mentioned, as shown in Table 3.

**Table 3:** Downloadable information on local elections for large non-capital municipalities.

CCAA	Municipality
Andalucía	Jerez de la Frontera
Principado de Asturias	Gijón
Catalunya	Hospitalet de Llobregat Sabadell Terrassa
Galicia	Vigo
Comunidad de Madrid	Alcalá de Henares Fuenlabrada Getafe Móstoles
Región de Murcia	Cartagena
Comunitat Valenciana	Elche

Source: compiled by the authors.

The variables, as well as the spreadsheets included in each of the workbooks, are the same as in the other elections, with the exception that here the last spreadsheets refer to the municipality; provisional and official.

#### European elections

In the European Parliamentary elections, the results spreadsheets of the Excel workbooks cover the range from ballot box to the geographical area of the CCAA and are offered, as mentioned in Methods, in two versions. One file shows the votes of the candidacies presented and another with the parties that are part of the same national coalition. In the merged data workbooks, the CANDIDADURAS spreadsheet has a structure similar to that of a general election (see Fig. 1). As an example, Table 4 shows the candidacies for the 1987 elections that were presented under the same joint denomination with different names in different regions.

**Table 4:** Example. Groupings of candidacies in the European Elections of 1987.

Group	Names of Candidacy
Coalición Izquierda de los Pueblos (CIP)	Esquerra dels Pobles-Entesa dels Nacionalistes (CIP.ENE), in Catalunya. Euskadiko Ezquerra-Izquierda de los Pueblos (EE.CIP), in Navarra and País Vasco. Esquerda dos Pobos PSG-EG (PSG.EG.CIP), in Galicia. PSM - Esquerra Nacionalista-Esquerra dels Pobles (PSM.EN.CIP), in Illes Balears. Coalición Izquierda de los Pueblos (CIP), in the rest of regions.
Izquierda Unida (IU)	Izquierda Unida-Iniciativa per Europa (IU.IPE), in Catalunya. Coalición Izquierda Unida (IU), in the rest of regions.
Por la Europa de los Pueblos (PNG)	Herrien European Alde-Europa de los Pueblos (EA.PNG.ERC) in País Vasco and Navarra. Per l’Europa de les Nacions (ERC.EA.PNG), in Comunitat Valenciana. Pola Europa dos Pobos (PNG.EA.ERC), in Galicia. Coalición por la Europa de los Pueblos (EA.ERC.PNG), in the rest of regions.
PSOE	Partit dels Socialistes de Catalunya (PSC.PSOE), in Catalunya. Partido Socialista Obrero Español (PSOE), in the rest of regions.

Source: compiled by the authors.

In the LEEME (README) spreedsheet of each of the merged files, the interested reader can consult the number of candidacies with different names that competed in the corresponding election and the number of groups of candidacies resulting from the merge of candidacies. Table 5 summarizes that information for all elections.

**Table 5:** Number of Candidacies and groups in European Parliamentary elections.

Year	Initial number of Candidacies	Number of groups
1987	44	35
1989	64	33
1994	58	35
1999	74	36
2004	72	31
2009	61	35
2014	70	39
2019	58	32

Source: compiled by the authors.

#### Other files

**Fig. 4:**
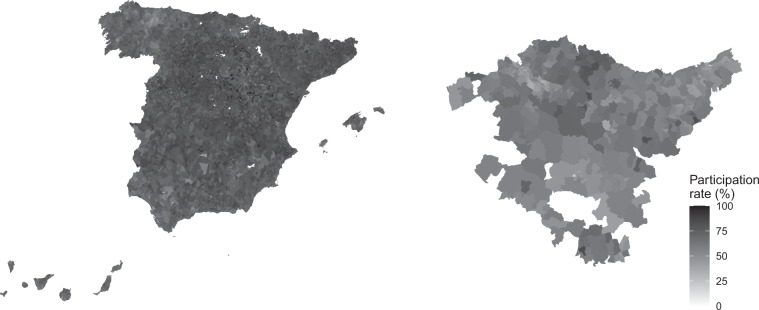
Cartography of the participation rate in the elections to the Congress of Deputies of April 2019 (left panel) and in the elections to the Basque Parliament of July 2020 (right panel).

Within the “Others” data set, sets of data are offered resulting from linking the electoral results with other types of information (for example, demographic or cartographic). Depending on the nature of the data, these are offered in Excel workbooks, which follow the same logic as indicated in the previous cases, or in other types of files. For example, in “Others” you can find the electoral cartography (for the scope of the census section) corresponding to the results of the elections to the Congress of Deputies of April 2019, and the results of the elections to the Basque Parliament (Eusko Legebiltzarra) held in July 2020. Such cartographies are offered in compressed files (rar) that contain all the geographic files necessary for their processing with appropriate software (e.g., ArcGIS, gvGIS, QGIS or R). The corresponding electoral results have been added to these files, through the associated dbf file. The LEEME.txt file contained in the rar files provides more details. Figure 4 shows the graphic representation of the participation rate registered in the two elections indicated above.

Due to its characteristics, the “Others” data set is the one with the greatest potential for future growth. In this sense, SEA is, and always will be, an unfinished project, as the possibilities of linking electoral results with other data sources are practically limitless. In terms of basic information, as well as adding to SEA the databases for the future electoral processes that will be held in Spain, one of our objectives is to complete those databases for which we do not have information for the most detailed levels of aggregation. Likewise, “Others” will also incorporate new workbooks obtained after matching results corresponding to different elections, estimating vote transfer matrices using ecological inference techniques, or associating new socioeconomic variables with electoral results. For the first task, the strategies proposed in Pavía and López-Quilez^37^ and in Pavía and Cantarino^38^ may be useful; for the second, the functions proposed in Lau *et al*.^39^ and in Pavía and Romero^40^; and, for the third, there are a multitude of socioeconomic variables, available at the census section level (such as income indicators, offered by the INE on its website within its section ‘Atlas of household income distribution’) which could be linked to election results. Another area of work on our agenda is related to obtaining electoral results prior to the Civil War. The provision of digitized data from that time would open up new study possibilities for interested historians.

## Technical Validation

SEA contains the results of provisional counts (usually available from small areas) and official results (only available at the aggregate level) of all the electoral processes held in Spain after the restoration of democracy. As validation we have checked the concordance (within a minimum margin of error) between the aggregations of the provisional results and the official ones.

Sometimes the provisional counts do not match the official ones. This can occur naturally for two reasons. Firstly, because the provisional files do not have the CERA vote (census of absent Spanish residents). Secondly, because there have been challenges to the results initially recorded, during the election night, that are later resolved and never corrected in the provisional scrutiny files. Further anomalies may arise due to a correction that we implement from GIPEyOP when faced with inconsistencies between variables or due to temporal inconsistencies observed for certain outcomes. Such interventions are collected and documented in the INCIDENCIAS spreadsheet; see example in Table 6.

**Table 6:** Example of data curation. General elections, April 2019 (in Spanish in the original).

**Error 1**	In province 11 (Cádiz), municipality 001 (Alcalá de los Gazules), district 01, section 001, ballot box B, in the CENSO + CERA column the value 590 appeared, with 295 being the value associated with CENSO.
**Action taken**	After detecting that the census had doubled, the CENSO + CERA column was assigned the value of 295.
**Error 2**	In province 22 (Aragón), municipality 235 (Torres de Alcanadre), district 01, section 001, ballot box U, the CENSO + CERA column had the value 176, with 88 being the value associated with CENSO.
**Action taken**	After detecting that the census had doubled, the value 88 was assigned to the CENSO + CERA column.

Source: compiled by the authors.

Given that the data offered in SEA correspond to the official provisional results, any modification of the raw data must be properly documented so that it can be reversed. We know that there are errors and misprints in the data, generally caused by small operational errors that any human activity entails. But these are often transferred to the results and proclaimed as final. The lack of incentive by political parties to request the revision of some misprints, even though they are aware of it, is often due to the fact that a correction may not have any practical consequence in terms of electoral results (distribution of representatives) and would have costs in terms of the legitimacy of the process.

The incidences that can be found in the databases are very varied. In our previous work, before considering undertaking the systematic organization of electoral results that SEA represents, we came across situations in which detected misprints persisted and were transferred to the official results. One example was seen in the results from a ballot box in which the votes received by the candidacies were shifted one column from a certain candidacy, as a consequence of a repetition or an omission counted as a zero. In these circumstances, the error can be dealt with in one of two ways; either leave the data as they are since they are official and have been proclaimed by the electoral authority as final, or correct the errors detected and document them so that the analyst can decide whether to work with the ‘correct’ data or with the official ones. At GIPEyOP we adopt this second approach.

Our future areas of work include systematically auditing all the existing databases in order to detect, correct and document all the errors and misprints that still exist in the provisional counting files. Thus, in the interest of this research group, any researcher who makes use of the files and detects any type of error should notify us so that we can reflect it as an incident. A very efficient way to detect errors in the data is by using the data. This will allow us all to have the best possible data to address future research. Documentation of the incidents detected and the actions implemented (see example in Table 6) is also essential. Having all the changes that are made to the ‘official’ data correctly documented is essential for allowing any changes introduced to be reversed, if deemed appropriate.

## Usage Notes

When using the SEA database or part of it, please cite this manuscript and the particular references related to the workbook being used. For any questions, suggestions or requests for collaboration regarding SEA please contact the corresponding author.

## Data Availability

Processing of raw data has been performed using ad-hoc scripts in the statistical software R, version 4.0.2 (R Core Team, 2020). Code is available on http://gipeyop.uv.es/gipeyop/base_datos/21_otros/CODIGO_SEA.zip.

## Online-only Table

**Online-only Table 1:** Sources in Autonomous Communities (CCAA) and their current status.

CCAA	Source/Status
Andalucía (AND)	Junta de Andalucía. Consejería de Justicia e Interior http://www.juntadeandalucia.es/justiciaeinterior/siel/extraccion.html Not active
Aragón (ARA)	Instituto Aragonés de Estadística https://aplicacionesportalaragon.aragon.es/tablas/iaest/areas-tematicas/15_sector_publico_elecciones/04_elecciones/elecciones-a-cortes-de-aragon.html Active (only at municipal level for 2007, 2011, 2015 and 2019). https://datos.gob.es/es/catalogo/a02002834-elecciones-a-cortes-de-aragon-ano-2011-detalle-mesa-electoral Active, polling station data 2011. https://datos.gob.es/es/catalogo/a02002834-elecciones-a-cortes-de-aragon-ano-2015-detalle-mesa-electoral1 Not active, polling station data 2015. https://resultados.elecciones.aragon.es/Mesas/es Not active, polling station data 2019.
Principado de Asturias (AST)	Sociedad Asturiana de Estudios Económicos e Industriales (SADEI) http://www.sadei.es/datos/sad/Eleccionesasp/eleccioneshtml.aspx
Canarias (CAN)	Instituto Canario de Estadística http://www.gobiernodecanarias.org/istac/jaxi-istac/menu.do?uripub = urn:uuid:94a26e66-9697-465e-a66e-0993795364d2 Active, from 1991 to 2015 for districts. 2019 data no longer available.
Cantabria (CANT)	Consejería de Presidencia y Justicia. Instituto Cántabro de Estadística (ICANE) https://www.icane.es/society/elections Active, for data from the 1983 to the 2007 elections, votes only. The 2019 information was obtained directly from the manager of the Socio-demographic Statistics Department.
Castilla La Mancha (CLM)	Junta de Castilla La Mancha https://elecciones2019.castillalamancha.es/resultados/descargar-los-resultados-de-las-elecciones-desglosado-por-mesas Active, results by polling station 2019. http://elecciones2015.castillalamancha.es/resultados/descarga-la-base-de-datos-de-resultados-definitivos Not active, results by polling station 2015. https://elecciones2019.castillalamancha.es/resultados/descargar-los-resultados-de-las-elecciones-autonomicas-anteriores-por-municipio Active, results by municipality for elections from 1983 until 2019.
Castilla y León (CyL)	Junta de Castilla y León https://datosabiertos.jcyl.es/web/jcyl/set/es/sector-publico/elecciones_2019/1284874794138 Active, data by polling station 2019 and by municipality for other elections.
Catalunya (CAT)	Generalitat de Catalunya https://resultats.dadeselectorals.gencat.cat/#/ Active, data by polling station since the elections of 1980 until 2021.
Comunidad de Madrid (MAD)	Dissemination of electoral results of the Community of Madrid (DECMA). https://www.comunidad.madrid/dcma/decma/P/areadescarga_list.php Active, by polling station since the elections of 1995 until 2019.
Comunitat Valenciana (CV)	Initially ABACUS and later the ARGOS Information Portal. Generalitat Valenciana http://www.argos.gva.es/ahe/cas/buscaEleccionesC.html Active, information by polling station for elections from 1995 until 2019.
Extremadura (EXT)	Presidencia de la Junta de Extremadura Available directly from the Administration department.
Galicia (GAL)	Xunta de Galicia https://abertos.xunta.gal/catalogo/administracion-publica/-/dataset/0426/eleccions-parlamento-galicia-resultados-2020 Active, data by polling station for elections from 2001 until 2020-
Comunidad Foral de Navarra (NAV)	Web elections of Navarra 2019 https://elecciones2019.navarra.es/Inicio/es Active, results by polling station 2019. http://www.navarra.es/home_es/Navarra/Instituciones/Elecciones + 2019/Resultados + Anteriores/ Active, but results by polling station are no longer available.
Balears, Illes (BAL)	Institut d’Estadística de les Illes Balears (IBESTAT) https://ibestat.caib.es/ibestat/estadistiques/societat/eleccions/2b95cdfb-7d12-4315-9b2c-0c8021b686ce Not active.
País Vasco (EUS)	Departamento de Seguridad. Gobierno Vasco https://www.euskadi.eus/ab12aAREWar/resultado/maint Active, results of all elections are available.
Región de Murcia (MUR)	Statistical Portal of the Region of Murcia https://econet.carm.es/web/crem/inicio/-/crem/sicrem/PU2077/sec. 0.html Active, only information by municipalities in 2015. https://datosabiertos.regiondemurcia.es/carm/catalogo/sector-publico/resultados-elecciones-autonomicas-2019-carm-datos-comunes-por-mesas-electorales Active, by polling station for 2019.
La Rioja (LRI)	Government of La Rioja https://www.larioja.org/estadistica/es/area-tematica-sociedad/procesos-electorales-rioja Active, by polling station for all the elections.
Ciudad Aut. de Ceuta (CEU)	Ministry of the Interior http://www.infoelectoral.mir.es/infoelectoral/min/areaDescarga.html Active, results available by polling station for all elections.
Ciudad Aut. de Melilla (MEL)	Ministry of the Interior http://www.infoelectoral.mir.es/infoelectoral/min/areaDescarga.html Active, results available by polling station for all elections.

Source: compiled by the authors
